# Editorial: African Cultural Models in Psychology

**DOI:** 10.3389/fpsyg.2022.844872

**Published:** 2022-02-14

**Authors:** Zewelanji N. Serpell, Vivian Afi Abui Dzokoto, Adote Anum, Faye Zollicoffer Belgrave

**Affiliations:** ^1^Department of Psychology, College of Humanities and Sciences, Virginia Commonwealth University, Richmond, VA, United States; ^2^Department of African American Studies, Virginia Commonwealth University, Richmond, VA, United States; ^3^Department of Psychology, University of Ghana, Legon, Ghana

**Keywords:** Africa, decolonization, cultural psychology, WEIRD and non-WEIRD societies, bottom-up approach

The overarching goal of **African Cultural Models in Psychology** is to consider how one might address an important gap in mainstream psychological research: insufficient inclusion of African perspectives in a field of study that largely represents Western hegemony. The United Nations estimates that Africa will make up a quarter of the world's population by 2050. Yet, mainstream Psychology's address of basic psychological processes has mostly developed without adequate representation from African samples. There have been limited efforts to better represent Africa in Psychology. The vast majority of existing work in African Psychology has been very applied in nature and designed to address problems that contemporary African societies face. Such work has contributed minimally to theorizing that informs the broader discipline. While there is recognition—at least by African Psychologists—of what psychology can do for Africa, there is not a similar appreciation for what Africa can do for Psychology. Thus, while there is discourse about the need to Africanize Psychology on the continent, there is no similar discourse to better represent Africa in Psychology to make the discipline less WEIRD (Henrich et al., 2010).

**African Cultural Models in Psychology** represents an effort to feature African research [see Ratele's (2017) typologies of African psychologies] that contributes to theory development in psychology and highlights African cultural models of the ways in which people exist within, experience, make sense of, interact with, and shape the world. Our vision for this special topic was a collection of original, data-driven research reports (utilizing quantitative, qualitative, and mixed method approaches), review papers, and theoretical papers grounded in research findings, with a focus on the first since previous work (e.g. Abubakar and van de Vijver, 2017) has done an excellent job of articulating review and theoretical perspectives. We were uniquely interested in papers using primarily bottom-up empirical (e.g. cultural indigenous, cultural practice) approaches, rather than top-down (e.g. cross-cultural validation) ones.

We made a concerted effort to invite as many Africa-based and Africa-focused psychologists as possible, making sure not to restrict our invitations to Anglophones. We used our professional networks and scoured websites of African departments to create email lists of potential contributors. We also explored publication outlets (journals and books) that focused on psychology in Africa and contacted individuals whose approaches in previous work were consistent with our goals. In all, we contacted a total of 466 researchers. Of these, 318 were unresponsive and 56 declined our invitation. We received 23 abstracts and rejected 5 proposals for perceived lack of fit. Of the remaining 18 potential submissions, we received 10 full manuscripts - 7 of which advanced through the peer review process. The response to our call for papers was negatively impacted by the global pandemic, which also contributed to a protracted turnaround time in the review process. Other barriers emerged; one that is noteworthy, given the purpose of this special issue, is publication costs. For some authors publication costs were prohibitive, even after a discount. This necessarily restricts who can publish work in high impact outlets like *Frontiers* to those who can afford it; which is highly problematic and impedes efforts to address the WEIRD problem in Psychology.

The resultant collection of 7 papers highlight African cultural models in Psychology, with dominant representation from Ghana and South Africa, a narrow representation—we admit—of Africa's diverse contexts. Rejecting Western models of cognitive abilities as universal, Oppong presents an African model of cognitive abilities developed from a systematic review of the literature on cognitive abilities using African samples. His model posits that general intelligence within the African context comprises three interconnected elements: cognitive competence, wisdom, and socio-emotional competence. The study by Esiaka et al. shows evidence of cultural-differences in prioritization of relations (e.g., mother vs. spouse) in an eldercare dilemma (institutionalization vs. integration in home). For example, Ghanaian participants expressed more disapproval for institutionalization than their American counterparts. The qualitative study by De Smet and Boros examines how power distance, gender dynamics, and tightness norms impact the implementation of a Western-based Non Governmental Organization (NGO) intervention conducted in a specific agro-centered initiative in a rural area in Ethiopia. Hill et al. examined the structure and fit of two translated (Tshivenda and Southern Sotho) versions of the South African Personality Inventory (SAPI). The language in which the inventory was developed influenced the factor loadings, especially for Neuroticism. Further, Traditionalism-Religiosity displayed poor factor loadings in both translated versions. The empirical paper by Wissing et al. provides evidence for global shifts in individualism-collectivism cultural patterns by highlighting the continuing prevalence in South Africa and Ghana of traditional African grounding of psyche as interdependent, but also as counterintuitively independent, hinting at an emerging shift in cultural constructions of experience. In their paper exploring the construct of wellbeing, Osei-Tutu et al. found a relationship between indigenous Ghanaian and hegemonic perspectives of the construct. Analysis of interviews in four Ghanaian languages indicated that some features of local models, such as positive affect and good physical health, mirrored hegemonic psychological science perspectives. However, local understandings of wellbeing also foregrounded moral living, material success, proper relationality, and peace of mind, suggesting a maintenance orientation to wellbeing. De Man et al. found that among people at risk for or with Type 2 diabetes in a South African Township, healthy diet behavior was positively associated with autonomous motivation (perception of support and competence) and negatively associated with controlled motivation (societal pressure, shame). Autonomous motivation mediated the effect of perceived competence and relatedness on healthy eating. These 7 papers are almost all empirical, and all demonstrate how harnessing African cultural models expands our understanding of the human experience.

Our aim in housing this project in an open access resource was to encourage attention to data-driven theory development from the African continent even as Psychology is applied in practice to African settings. Each paper in this special issue uniquely contributes to this agenda by challenging prevailing theories, and raising important questions about cultural dimensions of psychological phenomena. Our hope is that in highlighting the work of African researchers, we have opened the door to future research projects and collaborations for psychologists interested in making psychology less WEIRD.

## Author Contributions

ZS and VAD co-wrote the initial draft. ZS, VAD, AA, and FB edited the draft. All authors contributed to the article and approved the submitted version.

## Conflict of Interest

The authors declare that the research was conducted in the absence of any commercial or financial relationships that could be construed as a potential conflict of interest.

## Publisher's Note

All claims expressed in this article are solely those of the authors and do not necessarily represent those of their affiliated organizations, or those of the publisher, the editors and the reviewers. Any product that may be evaluated in this article, or claim that may be made by its manufacturer, is not guaranteed or endorsed by the publisher.
